# Oral Administration of Nano-Emulsion Curcumin in Mice Suppresses Inflammatory-Induced NFκB Signaling and Macrophage Migration

**DOI:** 10.1371/journal.pone.0111559

**Published:** 2014-11-04

**Authors:** Nicholas A. Young, Michael S. Bruss, Mark Gardner, William L. Willis, Xiaokui Mo, Giancarlo R. Valiente, Yu Cao, Zhongfa Liu, Wael N. Jarjour, Lai-Chu Wu

**Affiliations:** 1 Division of Rheumatology and Immunology, Wexner Medical Center at The Ohio State University, Columbus, Ohio, United States of America; 2 Department of Internal Medicine, Wexner Medical Center at The Ohio State University, Columbus, Ohio, United States of America; 3 Center for Biostatistics, Wexner Medical Center at The Ohio State University, Columbus, Ohio, United States of America; 4 College of Pharmacy, Wexner Medical Center at The Ohio State University, Columbus, Ohio, United States of America; 5 Comprehensive Cancer Center, Wexner Medical Center at The Ohio State University, Columbus, Ohio, United States of America; University Medical Center Freiburg, Germany

## Abstract

Despite the widespread use of curcumin for centuries in Eastern medicine as an anti-inflammatory agent, its molecular actions and therapeutic viability have only recently been explored. While curcumin does have potential therapeutic efficacy, both solubility and bioavailability must be improved before it can be more successfully translated to clinical care. We have previously reported a novel formulation of nano-emulsion curcumin (NEC) that achieves significantly greater plasma concentrations in mice after oral administration. Here, we confirm the immunosuppressive effects of NEC *in vivo* and further examine its molecular mechanisms to better understand therapeutic potential. Using transgenic mice harboring an NFκB-luciferase reporter gene, we demonstrate a novel application of this *in vivo* inflammatory model to test the efficacy of NEC administration by bioluminescent imaging and show that LPS-induced NFκB activity was suppressed with NEC compared to an equivalent amount of curcumin in aqueous suspension. Administration of NEC by oral gavage resulted in a reduction of blood monocytes, decreased levels of both TLR4 and RAGE expression, and inhibited secretion of MCP-1. Mechanistically, curcumin blocked LPS-induced phosphorylation of the p65 subunit of NFκB and IκBα in murine macrophages. In a mouse model of peritonitis, NEC significantly reduced macrophage recruitment, but not T-cell or B-cell levels. In addition, curcumin treatment of monocyte derived cell lines and primary human macrophages *in vitro* significantly inhibited cell migration. These data demonstrate that curcumin can suppress inflammation by inhibiting macrophage migration via NFκB and MCP-1 inhibition and establish that NEC is an effective therapeutic formulation to increase the bioavailability of curcumin in order to facilitate this response.

## Introduction

Diferuloylmethane (curcumin) is a natural polyphenolic compound derived from pulverizing the dried rhizomes of the turmeric plant (*Curcuma Longa*). Native to India and Southeast Asia, turmeric is widely used as a spice and yellow food coloring agent, but has also been used extensively for hundreds of years in Ayurvedic medicine to treat multiple ailments, such as allergies, asthma, sinusitis, rheumatisms, ulcers, trauma, diabetes, and inflammatory skin diseases [1]. Intriguingly, epidemiological studies from this region have shown a decreased prevalence and milder onset of rheumatoid arthritis and other connective tissue disorders when compared to the West [2]. Curcumin is the principle bioactive component of turmeric and has shown no dose-limiting toxicity in phase I clinical trials [3]. When combined with its safety for human use, this long history as an effective anti-inflammatory agent has resulted in a great interest recently in the exploration of the potential clinical application of curcumin in the prevention and treatment of a variety of inflammatory diseases [4].

Curcumin has been shown to interact with many molecular targets, which is demonstrated by data derived from studies examining type II diabetes, cardiovascular disease, asthma, inflammatory bowel disease, and arthritis. In diabetic rats, curcumin inhibited nuclear factor-κB (NFκB) and prevented the pathological elevation of interleukin (IL)-1β [5]. Mononuclear inflammation of aortic walls was reduced in a mouse model of cardiovascular disease with curcumin treatment and corresponded with lower tissue concentrations of IL-1β, IL-6, and monocyte chemoattractant protein (MCP)-1 as well as decreased NFκB DNA-binding [6]. Additionally, Toll-like receptor (TLR) 4-induced NFκB activation was inhibited by curcumin in an experimental model of colitis [7] and mild improvement of disease has been observed in several pilot studies in humans with inflammatory bowel disease [8], [9]. To examine the effects of curcumin in limiting the pathological inflammation observed in osteoarthritis, human chondrocytes were stimulated with IL-1β. These results demonstrated that curcumin suppressed IL-1β-induced NFκB activation by inhibition of IκBα phosphorylation [10]. Collectively, these results suggest that curcumin has a potent suppressive influence over inflammatory disease pathology.

Despite its use over centuries and the recent molecular data supporting the anti-inflammatory effects of curcumin, its clinical application to treat inflammatory disease is limited. The primary obstacle to this progress is the inadequate systemic bioavailability of orally administered curcumin in the current delivery systems, which has restricted therapeutic advancement for the treatment of disorders that are not localized to the gastrointestinal (GI) tract. Since curcumin is barely soluble in water, poor absorption is attained from luminal epithelial cells in the GI tract. In concordance, rats given an oral dose (1 g/kg) of curcumin excreted 75% in the feces unchanged, with less than 0.02% recovered from the liver, kidney, and body fat [11]. Moreover, the curcumin that is absorbed in the body is rapidly metabolized. Several studies analyzing plasma levels of curcumin or its metabolites after oral administration in humans have detected only small amounts in spite of very high doses. In healthy volunteers, no curcumin was detected in the serum with single doses of 0.5–8 g and low levels were detected at doses of 10–12 g [12]. Even with daily oral doses of 2–4 g for a month, free curcumin was undetectable and metabolic conjugate concentrations were very low (0.16 µM) in the plasma of colorectal cancer patients [13].

To enhance curcumin bioavailability by increasing solubility and by providing metabolic protection, we have recently developed and characterized a novel formulation of nano-emulsion curcumin (NEC) that has a higher loading capacity than other oral delivery systems [14]. This formulation makes curcumin more systemically available and protects it from metabolism following GI tract absorption, which results in a 10.5-fold increase in bioavailability relative to curcumin alone. Here, we use NEC therapeutically in mouse models of acute inflammation and identify a unique influence of curcumin over macrophage migration. Following lipopolysaccharide (LPS) stimulation, oral administration of NEC was found to significantly reduce levels of MCP-1 and macrophage accumulation in peripheral blood. Additionally, macrophage recruitment was suppressed in thioglycollate-induced peritonitis with NEC treatment, but had no significant influence over levels of B-cells or T-cells. Macrophage migration was also inhibited *in vitro* in primary human macrophages and in cell lines of both murine and human origins. These results establish a novel function of curcumin in selectively inhibiting macrophage-mediated inflammation and validate NEC as a candidate drug for future studies as an anti-inflammatory agent.

## Materials and Methods

### Mice

BALB/c mice were purchased from the Jackson Laboratories (Bar Harbor, ME) and BALB/C-Tg(NFκB-RE-luc)-Xen mice carrying a transgene containing six NFκB responsive elements and a modified firefly luciferase cDNA were purchased from Caliper Life Science (Hopkinton, MA). All animals were housed at The Ohio State University Wexner Medical Center (OSUWMC) in a BSL-3 barrier facility. Standard housing conditions included a 12 h light/dark cycle with chow and water available *ad libitum*. Facilities were maintained at 22–23°C and between 30–50% relative humidity. All animal maintenance and protocols were specifically approved for this study by the Institutional Animal Care and Use Committee through The University Laboratory Animal Resources at OSUWMC.

### Human Samples and PBMC Isolation

Healthy volunteers were recruited for the study from local communities and The American Red Cross. Participation was through an approved Institutional Review Board (IRB) protocol at OSUWMC. All participants in this study provided written consent that was documented and kept on permanent file per IRB policy at OSUWMC. All materials and procedures associated with patient consent were preapproved within IRB protocol at OSUWMC. This study was specifically approved through the IRB at OSUWMC. The samples were obtained as whole blood or filtered blood samples and PBMCs were isolated using Ficoll (GE Healthcare, Pittsburgh, PA) as previously described [15].

### Cell Culture

Curcumin powder (Acros, Organics, NJ) was dissolved in dimethyl sulfoxide (DMSO; Fisher Scientific, Pittsburgh, PA). THP-1; Jurkat, Clone E6-1; and mouse RAW 264.7 cells were cultured in complete RPMI medium containing 10% FBS (Life Technologies, Grand Island NY) according to previously described methods [15]. Cells were maintained in logarithmic growth phase and incubated at 37°C/5% CO_2_ before treatment with or without the indicated concentrations of curcumin and/or ultrapurified LPS from *E. coli* Serotype EH 100 (Sigma-Aldrich, St. Louis, MO). Equal volumes of DMSO were used in final curcumin dilutions and in untreated, vehicle controls. After the indicated time periods, cells were harvested, washed in phosphate buffered saline (PBS; Amresco, Solon OH), and collected for further analysis as described below.

### THP-1 Differentiation and Monocyte-Derived-Macrophage (MDM) Culture

THP-1 cells were plated at a density of 1×10∧5 cells/mL and differentiated by treatment with 15 ng/mL phorbol 12-myristate 13-acetate (PMA; Sigma-Aldrich) for 4 days to induce macrophage differentiation and attachment. MDMs were isolated from human blood samples using an IRB approved protocol as previously described [15]. Cells were plated at a density of 6×10∧6 cells/mL and incubated overnight. Subsequently, the cell plates were washed with PBS and adherent cells were used to perform *in vitro* scratch assay as described below.

### *in vitro* Scratch Assay

A straight line was introduced within cell monolayers with a 200 µL or 1000 µL pipette tip and cell debris was removed by gentle washing with PBS. Cells were treated with experimental doses of curcumin and photographs were taken at various time points at 40 X or 80 X magnification using a Nikon TMS-F inverted research phase contrast microscope (Scopeoptic, Abingdon VA). Cells were gently washed with PBS before images were captured.

### NEC and Suspension Curcumin (SC) Preparation

NEC and SC were prepared as described previously [14]. Briefly, Curcumin powder (1.65 g; Acros, Organics, NJ) was dissolved in 10 mL polyethylene glycol (PEG) 600 (Sigma-Aldrich) by stirring with a magnetic bar and heating in a boiling water bath to 100°C for 15 minutes in a Falcon tube covered by aluminum foil until completely dissolved. After cooling the sample to room temperature for 15 min, 5 mL of Cremophor EL (Kolliphor; Sigma-Aldrich) was added and mixed by inversion. The NEC stock solution (100 mg/mL) was stored at room temperature and kept in the dark, as we have previously demonstrated <5% of curcumin decomposed following 60 days of storage [14]. Vehicle was prepared in an identical manner, but without the addition of curcumin powder. SC was prepared in 1% carboxymethylcellulose water solution and was mixed well just prior to use.

### LPS Administration and NEC Treatment

Mice were treated with 1 g/kg NEC or vehicle by oral gavage 10 min prior to stimulation with 2 mg/kg LPS via intraperitoneal injection. Vehicle controls were prepared simultaneously with NEC. Bioluminescence was measured using the *in vivo* imaging system (IVIS). Whole blood was collected via submandibular bleeding or from the subclavian artery at indicated time points. Leukocytes were purified for flow cytometry and serum was analyzed by enzyme-linked immunosorbent assay (ELISA) as described below.

### Thioglycollate-Induced Peritonitis and NEC Treatment

Mice were pretreated with 1 g/kg/day of NEC or vehicle for 5 days by oral gavage before intraperitoneal injection of thioglycollate (3 mL; 3% w/v; BD Difco, Franklin Lakes, NJ). Following 6 days of continued oral administration of 1 g/kg/day NEC or vehicle, peritoneal cells were isolated in PBS by intraperitoneal syringe collection for analysis by flow cytometry.

### *in vivo* Bioluminescent Imaging

BALB/C-Tg(NFκB-RE-luc)-Xen mice were given 150 mg/kg luciferin [Gold Biotechnology, Inc., St. Louis, MO; 15 mg/mL in PBS (pH 7; unadjusted)] through intraperitoneal injection and bioluminescent signals were captured following indicated time periods using IVIS 200 (Caliper Life Sciences). Data were quantitatively analyzed using IVIS Living Image software (v3.2).

### Flow Cytometry

Leukocytes were purified from blood using red blood cell lysis solution (eBioscience, San Diego CA) following manufacturer's protocol. Peritoneal cells or leukocytes were labeled with antibodies for anti-mouse CD3 (eBioscience), CD4 (eBioscience), CD8 (eBioscience), B220 (BD Biosciences, San Jose CA), F4/80 (eBioscience), TLR2 (eBioscience), TLR4 (R&D Systems, Minneapolis MN), or RAGE (R&D Systems) following manufacturer's protocol. THP-1 and Jurkat, Clone E6-1 cells were grown in complete RPMI medium at a density of 2×10∧5 cells/mL, supplemented with 1 µM, 10 µM, 20 µM, or 30 µM curcumin, and collected after 10 min or 24 h. Data were collected on the BD FACS Calibur platform (BD Biosciences) using CellQuest Pro (v5.1, BD Biosciences) and exported for analysis via FlowJo (v.7.6.5; Tree Star, Inc, Ashland, OR).

### ELISA

Serum was isolated from mice by centrifugation after allowing whole blood to clot at room temperature. ELISA was performed on diluted samples using the mouse CCL2 (MCP-1) Ready-SET-Go! ELISA reagent set (eBioscience) according to manufacturer's protocol. Absorbance values were measured by the Dynex MRX-TC Revelation microplate reader/colorimeter (Dynex Technologies, Chantilly, VA) at 450 nM wavelength. Protein concentrations were determined with reference to a serial dilution of recombinant mouse MCP-1 protein (eBioscience). Linear equation for the standard curve and sample analyses was generated by exporting results to Microsoft Excel (version 2010).

### Western Blotting

Western blotting was performed as described [15]. Antibodies used in this study: p(Ser 536)-p65, p-IκBα, NOS2, and β-actin (Santa Cruz Biotechnology Inc., Dallas, TX); MCP-1 (Cell Signaling Technology, Beverly MA). Signal intensities were measured with ImageJ software (v1.45s; NIH, Bethesda, MD) and analyzed with Microsoft Excel (version 2010). Quantitations of protein expression were determined by normalization to β-actin and corresponding fold differences were related to time zero expression levels.

### Nitrite Assay

RAW 264.7 cells were stimulated with LPS (100 ng/mL) with or without curcumin at various concentrations for 24 h. Nitrite assays were performed using conditioned culture medium and the Griess Reagent System (Promega, Madison WI) according manufacture's protocol.

### Cell Viability Assay

Cell viability was measured using the AQueous One Solution cell proliferation assay according to the manufacturer's protocol (Promega Corporation, Madison, WI). Cells were treated with the indicated concentrations of curcumin and compared to DMSO vehicle controls. Absorbance values at 490 nM wavelength were recorded with the Dynex MRX-TC Revelation microplate reader/colorimeter (Dynex Technologies). The absorbance value without curcumin treatment was designated +1 in RAW 264.7 cells and the relative fold change in viability was determined for various treatments after normalizing to background absorbance levels. Absorbance values at baseline were designated +1 for THP-1 cells and primary human macrophages.

### Statistics

All numerical data were expressed as mean values ± standard deviation. Statistical differences were determined by paired, two-tailed, Student *t* tests using Microsoft Excel (version 2010) and considered statistically significant when p≤0.05.

**Figure 1:**
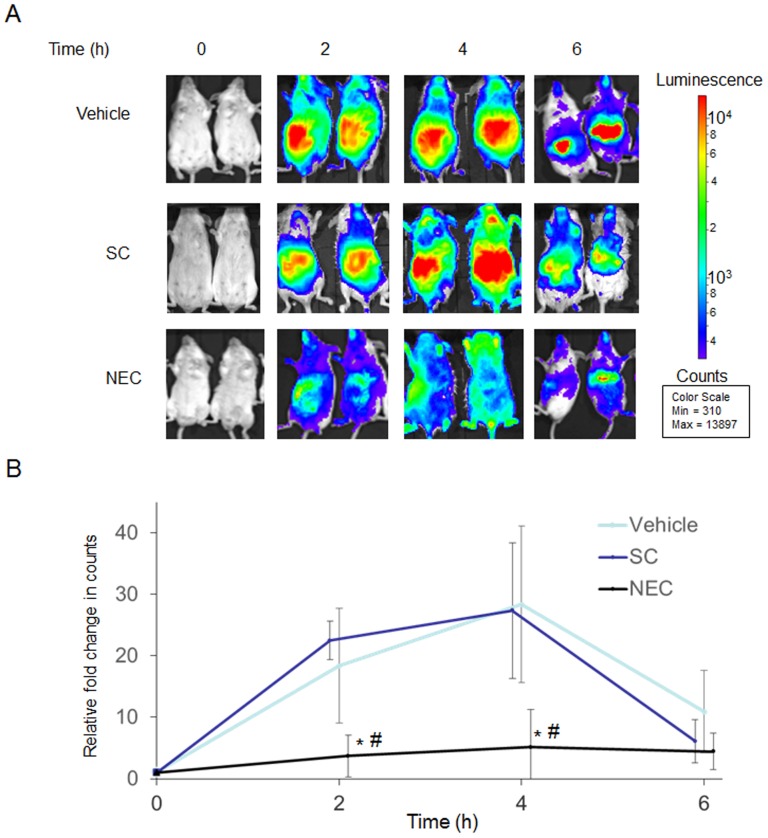
LPS-induced NFκB reporter gene expression is suppressed with nano-emulsion curcumin (NEC) in mice when compared to equivalent concentration of suspension curcumin (SC). Transgenic BALB/C-Tg(NFκB-RE-luc)-Xen mice (*n* = 13) were treated with 1 g/kg NEC, equivalent component concentrations of the nano-emulsion vehicle, or SC by oral gavage 10 min prior to LPS injection (IP, 2 mg/kg), and imaged (IVIS 200; 150 mg luciferin/kg IP) at 0 h, 2 h, 4 h and 6 h. The whole animal, including areas covering the thymus, lymph nodes, and the abdominal region were outlined and subjected to relative photon counting. **A,** Representative whole body bioluminescent images; **B,** Digitization of emitted light photons. The fold change in luminescent intensity is expressed relative to the photon counting in the mice at 0 h. Data were analyzed using the mixed effect model, as detailed in methods. *  =  *p*<0.0001 versus SC. #  =  *p*<0.0001 versus vehicle. IP  =  intraperitoneal.

Bioluminescent imaging analysis (Figure 1) was carried out using the mixed effect model, incorporating repeated measures for each mouse [16]. Multiplicities were adjusted by Holm's method to control the family-wise error rate at 0.05 [17]. SAS9.3 software (SAS, Inc; Cary, NC) was used for analysis.

## Results

### Bioluminescent Imaging Shows That NEC is Superior to Curcumin Alone in the Suppression of LPS-Induced NFκB Activation

While enhanced bioavailability has been shown previously [14], the effectiveness of our novel NEC formulation as an oral anti-inflammatory agent was not characterized. Since curcumin has been shown to inhibit NFκB DNA-binding [6] and expression [5] in animal models of inflammatory disease, we used transgenic mice with an NFκB-driven reporter gene to assess the ability of NEC to modulate inflammatory responses compared to vehicle controls or curcumin alone (SC) suspended at the same concentration. BALB/C-Tg(NFκB-RE-luc)-Xen mice, commonly called NFκB-RE-luc, have a modified firefly luciferase reporter transgene under the control of NFκB responsive elements. This mouse model has been used to study the transcriptional regulation of NFκB in inflammatory responses and is quantified by *in vivo* imaging systems (IVIS) that measure emitted bioluminescence. Thus, the digitized light photons counted after luciferin injection correspond to luciferase activity and are correlative with NFκB transcriptional activation.

NFκB-RE-luc mice were pre-treated with 1 g/kg NEC, an equivalent amount of the nano-emulsion vehicle, or suspension curcumin (SC) by oral gavage and challenged with 2 mg/kg LPS 10 min later. Luciferase activity was measured by whole body IVIS imaging following intraperitoneal luciferin injection and bioluminescent signals were counted as emitted photons relative to baseline levels in longitudinal analysis. Oral administration of NEC significantly reduced whole body bioluminescent signals with LPS stimulation at 2 h and 4 h relative to SC at the same concentration and vehicle controls (Figure 1A). NFκB activation was suppressed relative to vehicle 5-fold at 2 h (*p*<0.0001) and 5.5-fold at 4 h (*p*<0.0001) with NEC treatment. Similarly, photon counts decreased by 6-fold at 2 h (*p*<0.0001) and 5.3-fold at 4 h (*p*<0.0001) when compared to SC. These results show that our novel formulation of NEC has significantly more *in vivo* potency than curcumin alone and can effectively suppress LPS-induced NFκB activity, as measured by whole body bioluminescent imaging.

### NEC Inhibits Macrophage Accumulation in Blood

**Figure 2:**
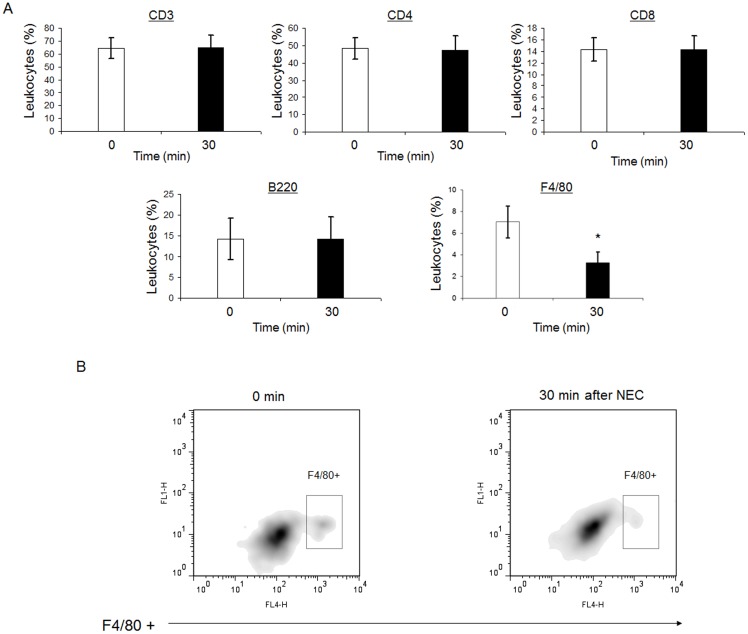
FACS analyses show that NEC selectively diminishes levels of blood monocytes. Leukocytes were isolated from whole blood collected from BALB/c mice (*n* = 6) before and 30 min after oral administration of NEC (1 g/kg). **A**, FACS analyses of leukocytes measuring the expression of cell surface markers: T-cells (CD3, CD4, or CD8), B-cells (B220), or monocytes (F4/80). * *p*≤0.05 versus time zero. **B**, Representative FACS analysis of F4/80^+^ expression.

While the molecular signaling of curcumin in cells and its effects as an anti-inflammatory mediator *in vivo* have been previously investigated, studies examining the differential effects of curcumin on immune cell subtypes in modulating these responses have remained largely unexplored. To assess whether curcumin can differentially influence immune cell subtypes, BALB/c mice were orally administered with NEC; purified leukocytes were isolated from whole blood after 30 min and analyzed by flow cytometry. Markers for T-cells (CD3, CD4, and CD8), B-cells (B220), and macrophages (F4/80) were used in flow assisted cell sorting (FACS) analyses to measure levels of each subtype. When compared to baseline measurements, CD3^+^, CD4^+^, CD8^+^, and B220^+^ cells were unchanged, but levels of F4/80^+^ cells were significantly reduced by 2.2-fold (*p*≤0.01) with NEC treatment (Figure 2A). FACS plots of the total leukocyte population also showed NEC-mediated reduction of F4/80^+^ macrophages in blood circulation (Figure 2B).

**Figure 3:**
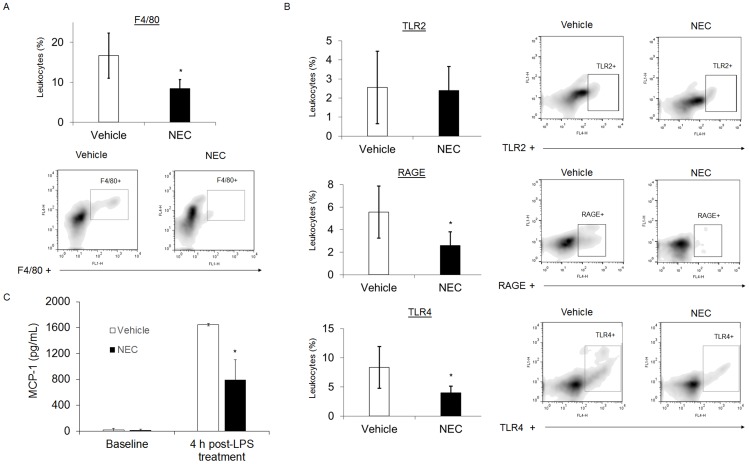
NEC suppresses LPS-induced TLR4 and RAGE expression in addition to blood monocyte accumulation. BALB/c mice (*n* = 10) were treated with NEC (1 g/kg) or vehicle by oral gavage and injected with LPS (IP, 2 mg/kg) 10 min afterward. At 4 h, whole blood was collected and leukocytes were isolated for flow cytometry. **A**, Quantitation (top) and representative images (bottom) from FACS analysis to measure F4/80^+^ cells. **B**, FACS analysis measuring TLR2, RAGE, or TLR4 expression on the surface of cells. **C**, ELISA of MCP-1 expression from mouse serum. *  =  *p*≤0.05 versus vehicle. IP  =  intraperitoneal.

To examine the influence of NEC on macrophage accumulation in the context of an acute inflammatory response, we stimulated wild-type BALB/c mice with LPS and blood was collected for FACS analysis after 4 h. In agreement with the data shown in Figure 2, oral administration of NEC diminished blood accumulation of F4/80^+^ cells (4-fold; *p*≤0.05) in the total leukocyte population with LPS stimulation (Figure 3A).

### NEC Downregulates TLR4 and RAGE Expression and Inhibits MCP-1 Secretion

Considering that curcumin has been shown to inhibit TLR4 expression with LPS stimulation in vascular smooth muscle cells [18] and inhibit NFκB activity resulting from TLR4 signaling [7], we also examined cell surface expression of TLR4, TLR2, and receptor for advanced glycation end products (RAGE) in LPS-stimulated mice. In addition to TLR4, LPS can also bind to TLR2 [19] and RAGE [20] to trigger pro-inflammatory NFκB signaling. While no effect was observed in the expression of TLR2 by FACS analysis, oral administration of NEC prior to LPS stimulation significantly reduced TLR4 expression on the surface of leukocytes by 4-fold (*p*≤0.05) and RAGE levels by 3-fold (*p*≤0.05) when compared to vehicle controls (Figure 3B).

Since MCP-1, one of the key chemokines regulating the migration and recruitment of monocytes/macrophages, is an NFκB target gene whose expression has been shown to be suppressed *in vivo* with curcumin administration [6], [21], [22], we also measured MCP-1 levels in the serum of these mice. ELISA showed minimal levels of MCP-1 at baseline, which was increased substantially after stimulation with LPS for 4 h (Figure 3C). In addition, NEC administration inhibited LPS-induced secretion by 2.1-fold (*p*≤0.01) relative to vehicle-treated control mice (Figure 3C). These data demonstrate that oral administration of NEC suppresses receptor expression upstream and inhibits MCP-1 production downstream of NFκB signaling in mice.

### Activation of NFκB is Rapidly Inhibited by Curcumin in Mouse Macrophages

**Figure 4:**
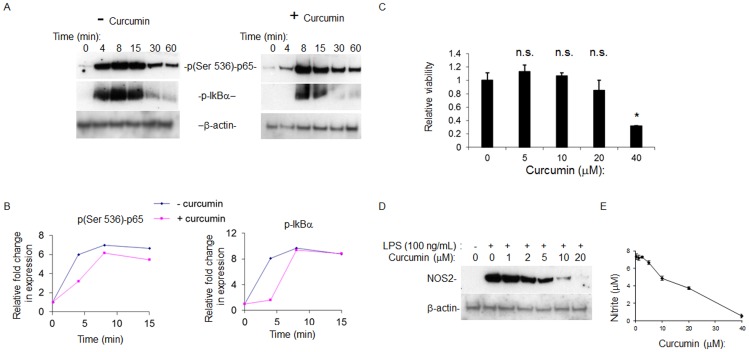
Curcumin suppresses LPS-induced phosphorylation of the p65 subunit of NFκB and IκBα. **A**, RAW 264.7 cells were stimulated by LPS (100 ng/mL) with or without 2 h pre-incubation of curcumin (final concentration of 20 µM) and harvested at the time points indicated for comparison to untreated controls containing equivalent volumes of DMSO. Western blot was performed with total cell lysates using p-p65, p-IκBα, and β-actin antibodies. **B**, Signals were quantitated by ImageJ and expressed as fold changes relative to time zero levels after normalization to β-actin. **C**, RAW 264.7 cells were stimulated with LPS (100 ng/mL) and increasing amounts of curcumin for 24 h. Cell viability assays were performed and the values are expressed relative to untreated vehicle controls. **D**, Western blot was performed with total cell lysates and antibodies against NOS2 and β-actin. **E**, Nitrite assays with the culture media in **(D)** using the Griess method. Experiments were performed in triplicate. *  =  *p*≤0.05 versus untreated control. n.s.  =  not significant versus untreated control.

To further characterize curcumin-mediated NFκB suppression in macrophages, RAW 264.7 cells were stimulated with LPS and treated with curcumin in a time course analysis. Previous work with this mouse macrophage cell line has shown that LPS stimulation of NFκB is inhibited by curcumin and leads to decreased inducible nitric oxide synthase (iNOS or NOS2) activity [23], [24]. Pae et al. demonstrated that curcumin suppressed NFκB activation by inhibiting both the p65 subunit and IκBα suppressor phosphorylation after treatment of RAW 264.7 cells for 4 h [24]. Since the direct regulation of this signal transduction pathway should occur rapidly *in vitro* and modulation of activation status at later time points may be attributable to indirect effects, we examined the effect of curcumin on NFκB activation immediately following LPS stimulation. Western blotting of total cell lysates to measure protein expression of phosphorylated p65 (p-p65) and IκBα (p- IκBα) revealed that curcumin treatment rapidly suppressed p-p65 and p-IκBα, but this effect was negligible after 30 and 60 min (Figure 4A). Using densitometry to quantitate protein signals after normalizing to loading controls, the difference between the relative fold changes in expression of p-p65 with curcumin treatment was 2.8-fold after 4 min, 0.8-fold after 8 min, and 1.2-fold after 15 min (Figure 4B). Additionally, the relative differences in the expression of phosphorylated IκBα were reduced by 6.5-fold after 4 min with curcumin, but only 0.34-fold and 0.03-fold after 8 and 15 min, respectively (Figure 4B).

To confirm that these observations were not due to curcumin negatively effecting cellular viability, MTS tetrazolium bioreduction assays measuring metabolically active cells were performed. RAW 264.7 cells were stimulated with LPS for 24 h and treated with increasing concentrations of curcumin. No significant reduction in cellular viability was observed with curcumin concentrations below 40 µM when compared to untreated cells (Figure 4C).

To show the downstream effect of curcumin inhibition of NFκB signaling, RAW 264.7 cells were stimulated by LPS with the addition of curcumin and total cell lysates were collected for protein analysis. NOS2 is not expressed in resting immune cells, but is activated by TLR4 signaling through NFκB after LPS stimulation to produce nitric oxide (NO), which is a key inflammatory mediator of innate immune responses [18]. Cells were treated with increasing concentrations of curcumin and lysates were collected to determine NOS2 expression. Additionally, conditioned media was used to measure nitric oxide production. While Western blotting showed no resting NOS2 expression, LPS stimulation resulted in an up-regulation after 24 h (Figure 4D). Furthermore, curcumin reduction of NOS2 expression was dose-dependent and undetectable levels were observed with curcumin treatment of 20 µM (Figure 4D). To verify reduced NOS2 activity in this experiment, NO levels were measured from the conditioned media using the Griess method. Relative to untreated levels, curcumin suppressed NO production by 10% with 5 µM, 44% with 10 µM, and 50% with 20 µM (Figure 4E). These results show that curcumin can rapidly suppress NFκB activity and inhibit the expression of key macrophage inflammatory mediators.

### Thioglycollate Elicited Peritoneal Macrophage Inflammatory Responses are Suppressed by NEC in Mice

**Figure 5:**
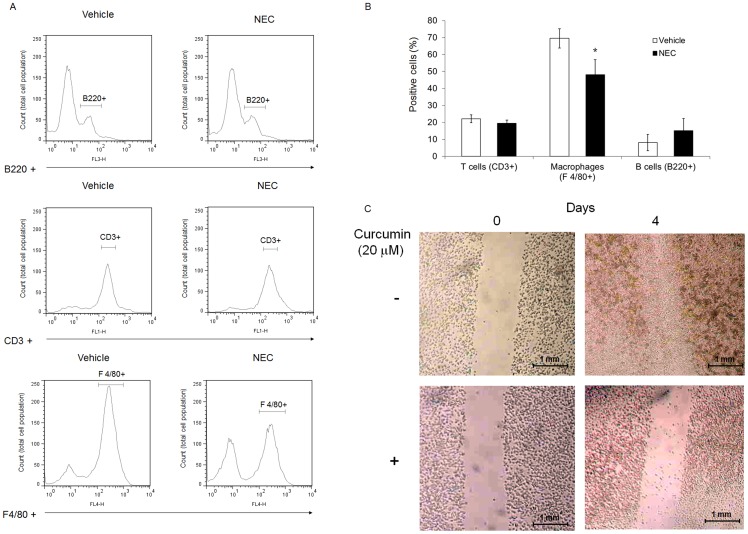
Curcumin inhibits mouse macrophage-migration both *in vivo* and *in vitro*. BALB/c mice (*n* = 6) were oral gavaged daily with vehicle or NEC (1 g/kg/day) for 5 days before intraperitoneal injection of thioglycollate (3 mL, 3% w/v) to induce inflammation. Following 6 days of continued oral administration of NEC or vehicle daily, peritoneal cells were isolated for flow cytometry. **A**, Representative images following FACS analysis to measure levels of B-cells (B220^+^), T-cells (CD3^+^) or macrophages (F4/80^+^). **B**, Cell subtype quantitations detected from total peritoneal cell isolates by FACS analysis. **C**, RAW 264.7 cells were plated to confluence and streaked. Cell migration was observed after 4 d with and without supplementing curcumin (20 µM) to the medium. Representative images taken from duplicated experiments. Original magnification X 80. *  =  *p*≤0.05 versus vehicle.

To examine the selectivity of NEC in suppressing macrophage mediated inflammation, we induced peritonitis in mice and measured immune cell subtype accumulation during the inflammatory response. Intraperitoneal injection of sterile thioglycollate medium in mice has been shown to induce an acute inflammatory response in the peritoneum. While the initial cells mediating the inflammation over the first 24 h consist primarily of neutrophils, macrophages predominate the peritoneal infiltrate after 3 days [25]. At later time points, T-cells and B-cells play important roles in the immune response, but are detected at lower levels [26]. Here, we used this model of thioglycollate-induced inflammation to evaluate the influence of NEC in suppressing a macrophage-mediated inflammatory response. BALB/c mice were pretreated with NEC (1 g/kg/day) via oral gavage for 5 days before injection of thioglycollate medium to elicit peritoneal inflammation. Subsequently, the mice were administered NEC for an additional 6 days and then intraperitoneal cavity cells were isolated for analysis by flow cytometry. Initial analysis revealed that NEC significantly suppressed the total number of cells isolated from peritoneal lavages by 1.76-fold (*p*≤0.02; data not shown) compared to mice treated with vehicle. Total peritoneal cell isolations were analyzed further by flow cytometry using antibodies to label B-cells (B220), T-cells (CD3), and macrophages (F4/80). FACS plots revealed no change in the B220^+^ or CD3^+^ populations, but a marked reduction was observed in the F4/80^+^ cells with NEC treatment (Figure 5A). Macrophage accumulation was reduced 1.44-fold (*p*≤0.02) when compared to vehicle-treated mice (Figure 5B). These data demonstrate that NEC can specifically suppress macrophage inflammatory responses *in vivo*.

### Curcumin Inhibits Cell Migration and Labels Human Monocytes

To demonstrate inhibition of mouse macrophage migration functionally, *in vitro* scratch assays were performed on RAW 264.7 cells with curcumin treatment. Adherent cells were cultured to near confluence and streaked to create a space to monitor cell migration. While untreated cells re-established a confluent monolayer after 4 days, curcumin treatment of 20 µM was able to reduce cell migration (Figure 5C).

**Figure 6:**
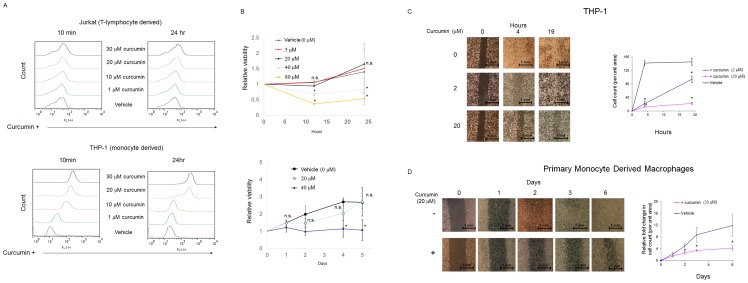
Curcumin labels monocytes and inhibits macrophage migration in human cells. **A**, Human cell lines Jurkat, Clone E6-1 and THP-1 were cultured in complete RPMI medium, supplemented with various amounts of curcumin or vehicle, and analyzed by FACS. Representative results are shown. **B**, THP-1 (top) and primary human monocyte derived macrophages (bottom) were treated with the indicated concentrations of curcumin and cell viability was measured over time by MTT assay. Values are expressed as fold changes relative to initial baseline levels. **C**, THP-1 cells were differentiated into macrophages and isolated by adherence to culture plates. Scratch assays were performed on adherent cells with or without curcumin to measure cell migration. Representative images (left) and migrated cell counts (right). **D**, Primary macrophages were isolated from healthy human blood samples (*n* = 4) and subjected to migration assays with and without curcumin (20 µM) for the indicated time. Representative images (left) and relative fold changes of migrated cells (right) are shown. Original magnification X 40. *  =  *p*≤0.05 versus vehicle. n.s.  =  not significant versus vehicle.

The molecular structure of curcumin exhibits detectable fluorescent properties that can be exploited to measure cellular binding and uptake [27], [28]. Here, curcumin was added to T-lymphocyte (Jurkat, Clone E6-1) or monocyte (THP-1) derived human cell lines and was detected by flow cytometry. Cells were treated with increasing concentrations of curcumin for 10 min or 24 h and washed before FACS analysis. Although Jurkat cells did not produce a detectable response with curcumin that differed from vehicle treatment at any concentration after 10 min or 24 h, THP-1 cells were labeled with curcumin in a dose-dependent manner at both time points (Figure 6A). FACS plots revealed that 10 min of curcumin treatment of THP-1 cells resulted in an enhanced signal at every concentration (Figure 6A). Furthermore, despite very modest increases at lesser concentrations, considerably higher signals were obtained with both 20 µM and 30 µM curcumin after 24 h (Figure 6A).

To examine whether curcumin-mediated suppression of macrophage migration also occurs in human cells, scratch assays were performed with THP-1 cells and primary human macrophages. Cellular viability was also measured with curcumin treatment in these cells to confirm that no significant cytotoxicity was observed at the concentrations used in these experiments. Relative to untreated THP-1 cells, 5 µM or 20 µM curcumin did not affect cell viability following 12 h and 24 h treatment, but a significant reduction was observed with higher concentrations of 40 µM and 80 µM (Figure 6B). Furthermore, 20 µM of curcumin similarly had no significant effect over cell viability in primary human macrophages isolated from healthy peripheral blood mononuclear cell preparations after treatment over 5 d relative to vehicle controls (Figure 6B).

THP-1 cells were differentiated to promote adherence and grown to confluence prior to streaking for migratory analysis by scratch assay. After washing away non-adherent cells and debris, 2 µM or 20 µM curcumin was added and cell migration was quantified as a cell count per unit area in the re-establishment of a confluent monolayer. After 4 h, untreated THP-1 cells migrated into the vacant space and completely filled the open gap (Figure 6C). When compared to cells treated with vehicle at the 4 h time point, 2 µM or 20 µM curcumin treatment significantly reduced migration by 84% (*p*≤0.004) and 92% (*p*≤0.001), respectively (Figure 6B). After 20 h, curcumin continued to inhibit cellular migration of THP1-cells by 35% with 2 µM (*p*≤0.001) and by 85% with 20 µM (*p*≤0.001) (Figure 6C). Similar assays were then performed on primary human macrophages isolated from healthy male and female subjects and showed that curcumin treatment also suppressed their migration over time when compared to vehicle-treated controls. After days 2, 3, and 6, migration was significantly reduced by 2.4-fold (*p*≤0.01), 5.3-fold (*p*≤0.025), and 7.6-fold (*p*≤0.025), respectively (Figure 6D). Collectively, these results show that curcumin can label monocytes and inhibit migration of macrophages in human cells.

## Discussion

Curcumin is the principle bioactive component of turmeric and has been used for centuries in Eastern medicine as an anti-inflammatory agent, which is the basis for recent interest in its potential therapeutic application. Although the suppression of inflammatory signaling has been characterized in recent years at the molecular level (reviewed in [4]), the clinical use of curcumin is not more widespread because of poor systemic bioavailability, rapid metabolism, and low solubility. Consequently, the most efficacious therapeutic progress with orally administered curcumin has been in the treatment of disorders localized to the GI tract, including inflammatory bowel disease [8], [9] and colon cancer [13]. To circumvent these physiological and biochemical obstacles in order to make curcumin more systemically available, we have developed a novel formulation of curcumin by nano-emulsification (NEC). Our previous pharmacokinetic and chemical analyses have shown that NEC can be delivered at a higher loading capacity with enhanced bioavailability and metabolic protection [14]. Here, our biological analysis of NEC confirms its immunosuppressive effects and demonstrates the influence of curcumin over macrophage migration.

Since curcumin has been shown to inhibit NFκB both *in vitro* [10] and *in vivo* [29], we used the NFκB-RE-luc mouse model to monitor the immunosuppressive capacity of NEC. Our results in Figure 1 show that with NEC treatment, LPS-induced NFκB activity was significantly reduced when compared to curcumin suspension. Using this model, the extent of *in vivo* NFκB activation was determined longitudinally in real-time by measuring the whole body bioluminescent reporter signal. While investigated previously, individual, organ-specific determinations of inflammation were not assessed due to the whole body bioluminescent imaging protocol and analysis performed in this study [30]. Considering the long list of curcumin formulations and delivery mechanisms with no clear candidate to move forward with clinically hitherto (reviewed in [31]), the application of this *in vivo* model could provide real-time feedback and allow therapeutic efficacy to be monitored in future drug screening or validation studies. Thus, we propose that our application of the NFκB-RE-luc mouse model is a novel system to investigate the therapeutic potential of NEC or other curcumin treatments, which enables a smaller cohort of mice to be used experimentally. This could also translate into less variable, more economical and efficient longitudinal analysis in the process of drug discovery.

While curcumin is known to suppress inflammation through inhibition of NFκB signaling, the influence over immune cell subtypes had yet to be characterized. When wild-type mice were treated with NEC (Figure 2), only the number of macrophages were reduced, which suggested a selective influence of curcumin on this immune cell subtype. Subsequent analysis in LPS-injected mice not only showed a similar suppression in macrophage accumulation, but also a significant downregulation of TLR4 and RAGE expression on the surface of these cells (Figure 3). These data suggest that the curcumin-mediated suppression of NFκB signaling with LPS treatment is due to downregulation of TLR4 upstream of NFκB. In concordance, downregulation of TLR4 expression on the surface of cells has previously been demonstrated with curcumin administration in microglial cells [32] and endothelial cells [33]. Similarly, a recent study by Fu et al. using an LPS-induced mouse model of mastitis reported reduced recruitment of inflammatory cells and inhibited TLR4 expression in breast tissue with curcumin treatment [34]. In addition to showing suppression of TLR4-mediated NFκB signaling, Fu et al. also found that curcumin inhibited LPS-induced phosphorylation of IκB. Using RAW 264.7 cells, we also observed a rapid decrease in LPS-mediated phosphorylation of IκB with curcumin treatment (Figure 4), which is supportive of an additional level of curcumin-mediated regulation of NFκB activity. Conceivably, suppression of NFκB signaling by NEC could account for decreased expression of transcriptional targets, including RAGE and MCP-1.

Data from the present study show that curcumin inhibits MCP-1 secretion and macrophage recruitment in the inflammatory process *in vivo*. Using a mouse model of acute peritonitis, macrophage recruitment was suppressed with oral administration of NEC, but T-cell and B-cell populations were not significantly affected (Figure 5). Accordingly, studies examining the influence of curcumin in kidney disease have shown attenuated macrophage recruitment in mouse models of LPS-induced renal inflammation [21] and immune complex-mediated glomerulonephritis [22]; both investigations also found that curcumin significantly reduced the expression of MCP-1. In addition to inducing monocyte chemotaxis to areas of inflammation and being expressed in a variety of cells [35], this chemokine is primarily regulated by NFκB [36]. Moreover, an atherosclerosis study recently showed that induction of MCP-1 expression in primary rat vascular smooth muscle cells was abrogated with curcumin treatment as a result of the inhibition of NFκB activation [18]. These data suggest that the selective inhibition of macrophage-mediated migration in the inflammatory process is likely attributable, at least in part, to curcumin-mediated suppression of NFκB transcriptional regulation of MCP-1 expression.

The results of our cell-based assays show that curcumin can also inhibit *in vitro* macrophage migration. Using mouse macrophages, curcumin treatment significantly reduced cell migration when compared to untreated cells (Figure 5). In human cells, suppression of migration was observed in a monocyte-derived cell line (THP-1) and in primary macrophages (Figure 6). After only 4 h of curcumin treatment of THP-1 cells, migration was significantly inhibited, which indicates that the response observed is indeed a reduction in cell migration and not due to an influence over proliferation. Additionally, since curcumin (20 µM) treatment did not have a significant effect over cell viability, toxicity is unlikely to be a confounding factor influencing these results (Figure 6). Recently, similar work has shown that curcumin can inhibit microglial migration and demonstrated that through inhibition of NFκB signaling, curcumin significantly modulated the microglial transcriptome to an anti-inflammatory and neuroprotective phenotype [32], [37]. Considering that microglial cells are the resident macrophages of the central nervous system, the anti-migratory/anti-inflammatory effects of curcumin on macrophages observed here suggest that this phenotype is produced by the altered transcription of many genes as a result of NFκB inhibition.

Since poor bioavailability is an issue with some commonly used pharmaceuticals, nano-emulsification should also have potential application beyond curcumin. For example, methotrexate (MTX) and levodopa (L-DOPA) are not readily absorbed by the GI tract, which leads to low systemic concentrations following oral administration. MTX is used in the treatment of rheumatoid arthritis and has been shown to be more efficacious in patients when its bioavailability was increased [38]. Similarly, L-DOPA has also shown effectiveness in the treatment of patients with Parkinson's disease using alternate delivery systems [39]. Moreover, co-administration of pharmaceuticals using nano-emulsification as a vehicle to deliver multiple drugs may be tremendously beneficial for therapeutics that have additive effects when administered in combinations. Accordingly, recent work has shown that co-treatment of tumor cells with MTX and curcumin had a synergistic effect [40]. The results of this study demonstrated that curcumin enhanced folate receptor expression allowing MTX to better target, enter tumor cells, and prevent thymine synthesis; thereby exhibiting its natural cytotoxic effect. Thus, it is reasonable to examine utilizing nano-emulsification to deliver safer, more efficacious doses of these and other drugs or drug combinations to their intended targets.

**Figure 7:**
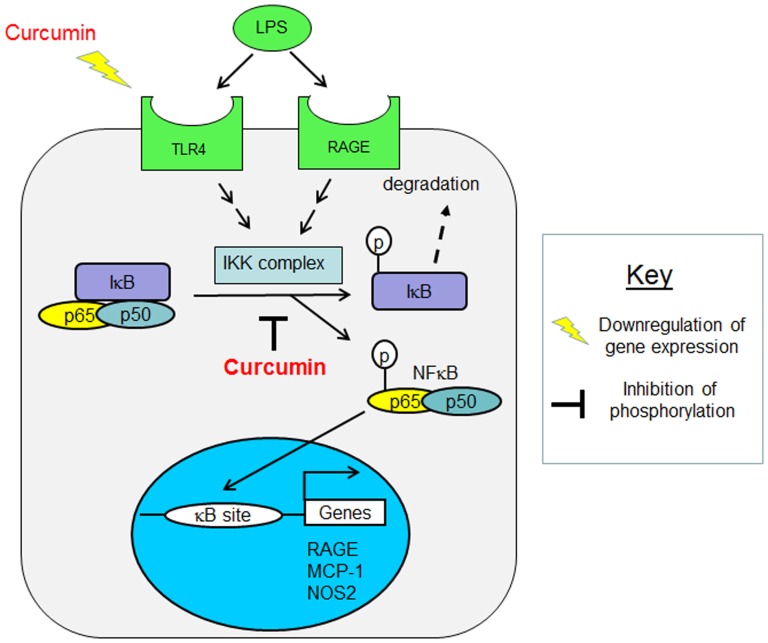
Schematic showing the cellular pathways by which curcumin (NEC) inhibits macrophage inflammatory signaling. Curcumin regulates inflammatory pathways at multiple levels: i) downregulation of TLR4 expression on the surface of cells and ii) inhibition of p65 and IκBα phosphorylation in the activation of NFκB. Thus, NEC inhibits transcriptional regulation *in vivo* of NFκB target genes, including RAGE, MCP-1, and NOS2, which leads to suppressed macrophage-mediated inflammatory responses.

Taken together, our studies have provided insight into the effects of curcumin in the suppression of inflammatory responses and suggest therapeutic applicability of NEC to a variety of inflammatory disorders. NEC was shown here to preferentially suppress macrophage recruitment and migration; our proposed schematic outlines the effects of NEC over macrophage inflammatory signaling based on the collective results of this study (Figure 7). Consequently, inflammatory diseases whose pathology is most strongly associated with macrophages may be optimal candidates for future NEC therapeutic studies; such diseases include glomerulonephritis, Crohn's disease, rheumatoid arthritis, inflammatory bowel disease, cardiovascular disease, diabetes, and obesity. Future work in our laboratory will be in testing the therapeutic potential of NEC in various models of chronic inflammatory conditions.
